# What characterizes a good mental health professional in court-mandated treatment settings?: Findings from a qualitative study with older patients and mental health care professionals

**DOI:** 10.1186/s40359-021-00624-4

**Published:** 2021-08-17

**Authors:** Helene Seaward, Tenzin Wangmo, Tobias Vogel, Marc Graf, Monika Egli-Alge, Michael Liebrenz, Bernice S. Elger

**Affiliations:** 1 Institute for Biomedical Ethics, University Basel, Bernoullistrasse 28, 4056 Basel, Switzerland; 2 Psychiatric Hospital of the University of Basel, Forensic Psychiatric Hospital, Basel, Switzerland; 3 Forensisches Institut Ostschweiz Forio, Frauenfeld, Switzerland; 4 Department of Forensic Psychiatry, Institute of Forensic Medicine, University of Bern, Bern, Switzerland; 5 Center for Legal Medicine (CURML), Medical Faculty, University of Geneva, Geneva, Switzerland

**Keywords:** Prison, Offender, Forensic, Involuntary, Coercion, Court-mandated treatment, Qualitative, Interview, Therapist-characteristics, Common factors

## Abstract

**Background:**

Therapist-related activities and characteristics such as empathy and genuineness are factors that significantly contribute to psychotherapy outcome. As they play a role in psychotherapy more generally, it can be expected that they are equally important in the treatment of court-mandated patients more specifically. At the same time, these treatment settings come with specific challenges—e.g. due to coercion and control—and it could thus be that some therapist-related characteristics might have a different empathy on the therapy. This interview study sought to investigate service providers’ and users’ perspectives on therapist-related characteristics in the context of detention.

**Methods:**

We conducted a qualitative interview study with 41 older incarcerated persons mandated to treatment, and 63 mental health professionals (MHP). The data analysis followed thematic analysis.

**Results:**

Patients and experts both emphasized the importance of treating patients with respect by taking a humanistic approach, that is, condemn the deeds but embrace the person and display genuine interest in supporting patients with any issue or concern that is of relevance to them. Furthermore, interviewees underscored that the coerciveness of the therapy context required to incorporate patients’ wishes into treatment planning, recognize and respond to the patients’ needs, and allow some choice within the given framework. Such inclusive attitude was deemed critical to engage and motivate patients to participate in treatment. In addition, it was emphasized that feedback and advice by the therapists need to be concrete, detailed and applied to each person’s current situation. Lastly, patients questioned MHP’s qualification when they did not progress in therapy.

**Discussion:**

Our findings indicate that some therapist-related activities and characteristics are of particular importance in court-mandated settings. These include genuine interest in the patient, a respectful and positive attitude, as well as the capacity to target sensitive issues in a directive but non-confrontational manner. Further research needs to identify specific expressions and behaviors that are linked to the aforementioned characteristics in the forensic context. Our study therefore contributes to much-needed empirical research on clinician and patient perspectives on therapist characteristics and activities in the treatment of court-mandated patients.

## Background

Therapist-related characteristics and activities are significantly associated with psychotherapy outcome factors [1–3]. They are amongst the common factors of psychotherapy, which shape a “theoretical model about mechanisms of change” overarching different psychotherapeutic methodologies [4, 5]. Empathy [6, 7], genuineness/congruence [8, 9], and positive regard [10] are the most reported therapist characteristics and activities. Evidence suggests that these characteristics are equally important in court-mandated treatment contexts, as they were linked to patients responding more positively to psychotherapeutic treatment and to enhancing their motivation to change [11–15]. Even though some implications of therapist characteristics might be common to all treatment conditions, others might be specific to the context [16, 17].

Court-mandated treatment settings come with several specificities for both patients and therapists. Persons who are mandated to treatment by a criminal court are not only diagnosed with a psychiatric disorder, but have also committed a crime, which is the justification for the official invasion of personal privacy and liberty. As a consequence, the overall purposes of mandated treatments are to reduce the risk to reoffend and to protect the public [18, 19]. Treatment goals are thus predefined and only indirectly centered on patient benefit. This situation differs considerably from general psychiatry and psychotherapy, where the treatment aims are to improve psychosocial functioning and ultimately the patient’s quality of life [20–22]. A further difference is that, therapists must focus on the person’s criminogenic factors in the context of court-mandated treatments. Moreover, as the referral by criminal courts guarantee treatment entry and participation, therapists might face resistance and lack of motivation because treatment is not requested by the patient [23]. Other important aspects that are specific to court-mandated treatment are the therapist’s dual role to care and control [24–27], limited medical confidentiality [28–31], as well as coercion [32, 33]. The precise impact of these aspects on psychotherapy process and outcome factors is unclear, but it is particularly important to understand their influence in court-mandated treatments [34]. This is because studies assessing the effectiveness of court-mandated treatments showed positive, negative, or equivocal effects on outcome measures such as criminal recidivism and psychiatric symptoms [34, 35]. These inconsistencies suggest that other intermediary factors—such as therapist characteristics—might play a role.

As the context of court-mandated treatments is characterized by coercion and control, evidence suggests that it is important to provide some degree of choice to encourage patients to actively participate in treatment [36]. Such recommendation is fostered, for instance, by transparent communication regarding treatment planning [37–39]. Patients want their views to be taken into account, they want to be treated as dignified and active actors, and to be involved in the dialogue on unmet needs and future goals [40–42]. Further, literature suggests that it is important for therapists to adopt a positive and respectful attitude towards patients. Feeling stereotyped or labelled is negatively linked to the development of trust and connectedness with mental health professionals [41–46]. Wittouck and Vander Beken [47] described this as “*‘us’ versus ‘them’ attitudes*”*,* and emphasized the importance of patients feeling valued and accepted. In the treatment of sex offenders, this seems to be of particular relevance, as they typically experience shame in relation to their offence [48]. This “*humane attitude”* [16] was further linked to mental health professionals being perceived as caring and supportive [47], such as when they showed genuine and authentic interest in the patient, took the time to talk and listen, took them seriously, and showed willingness to understand [37, 43, 45, 46, 45–46].

In addition, two recent reviews have concluded that a directive, authoritative but non-confrontational style is beneficial in the treatment of court-mandated patients [15, 32]. This should not be confounded with a dominant, authoritarian or confrontational-style, which was highlighted as detrimental in the therapeutic process. For instance, Marshall and Serran [13] conceptualized confrontation as a derogatory and aggressive communication style [11–14]. This is of particular importance when working with persons who committed an offence, as the justice system requires the crime to be targeted as part of treatment and often specifically links facilitation or relaxation of imprisonment to progress in openness to address past offenses. The way in which a mental health professional (MHP) shapes the conversation around these sensitive issues is therefore crucial.

Investigating in greater depth the way therapists should approach the specific setting of court-mandated treatment is important since, worldwide the number of persons mandated to treatment by court-order is rising [53]. Among them are older persons, whose proportion is growing faster than any other age group within the criminal justice system [54–56]. Within the Swiss prison context, these two groups overlap, as there has been a drastic increase of older persons sentenced to mandated treatment. For instance, the number of persons over the age of 49 sentenced under Swiss Criminal Code (SCC) article 59 (incarcerated persons mandated by court order to psychotherapeutic and psychiatric treatment) increased from 8.7% in 1999 to 17.8% in 2019 [57] and those sentenced under article 64 (indefinite incarceration) increased from 35.5 to 74.7% in the same time period [58]. Reasons to treat the group of older persons separately are, first, that response and effectiveness of treatments may differ in comparison to younger adults. Second, the higher prevalence of somatic health issues may challenge the application of psychotherapeutic interventions [59]. Particularly with regard to the older prison population it is important to note that the prevalence rates of physical and mental health problems are higher in comparison to younger persons in detention as well as in contrast to older persons living in the community [60, 61]. The fact that they are a population of high needs requiring intensive resources substantiates the need for research on particular therapy requirements for this age group. Also existing studies with older adults in the forensic context discuss service experiences of older persons [62–65], but they do not specifically investigate whether and how therapist characteristics and activities can impact on the therapy.

Evidence suggests that there are specific characteristics and activities needed to promote behavioural change in older patients legally referred to psychotherapeutic treatment. It is therefore crucial to shed further light into the particularities of correctional contexts to increase the effectiveness of mandated treatments. This qualitative interview study explores therapist characteristics and activities that patients and MHPs consider as beneficial in facilitating change in coercive treatment contexts. This study contributes to much-needed research on this topic to facilitate change in patients mandated to treatment by court order.

## Methods

This article follows the “Journal article reporting guidelines” for qualitative research by Levitt and Bamberg [66], which includes the COREQ-32 guidelines [67]. With the terminology *mandated treatment*, we refer to persons sentenced to punitive measures according to applicable criminal law (in the case of our participants, according to art. 56ss of the SCC) who are mandated to undergo psychotherapeutic treatment by means of a court-order (see the section on “context information” for further details). The methodology presented here is also described elsewhere; please see for example [30, 68].

### Study design

This qualitative study is part of a larger research project on mental health of older persons living in detention (‘Agequake in Prisons 2’, Swiss National Science Foundation [grant number *166043*]*.*). As part of the larger project, we not only gathered qualitative data from older persons in prisons and professional stakeholders (described below) but also quantitative information on older persons’ mental health condition from medical records and standardized surveys. As older persons in prison are a minority within the general prison population—although growing in certain settings (e.g. persons sentenced to measures)—there is little data on the mental health of this population [69], the overall goal of the qualitative data collection was to gain insights into their aging experiences in prison, living with a mental disorders, and their perspectives on prison mental health care. Ethics approval was obtained from the regional ethics committee (Ethikkommission Nordwest- und Zentralschweiz) which was followed by other local ethics committees. For Canadian expert-participants, approval was given by Correctional Service Canada.

### Study sites and participant inclusion criteria

We interviewed MHPs as well as patients receiving care in the prison setting. Patient-participants were recruited from Switzerland exclusively, while expert-participants from both Canada and Switzerland were included. Expert-participants were MHPs with work experience with detained patients (psychologists, psychiatrists, psychiatric nurses, social workers, occupational therapists). Canada and Switzerland were chosen as they both have a growing older incarcerated population. While key characteristics of this older population are likely to be similar—e.g. high prevalence rates of somatic and psychiatric illnesses—the way MHPs deal with these issues might differ. Thus, including professionals’ experiences in handling the older detained population from two countries could shed light into alternative care strategies. At the same time, it can highlight features that are common across differing jurisdictions, such as MHPs’ characteristics that older incarcerated person’s value during psychotherapy.

We included correctional institutions and forensic mental health facilities that housed adults, sentenced to long-term imprisonment (minimum of 3 years imprisonment, see Art. 10 SCC). We excluded correctional institutions that housed juvenile or remand prisoners exclusively as well as administrative detention centers (centers housing migrants for deportation). In Switzerland, prisons from the two major language regions (French and German speaking) were included, the Italian speaking language region was excluded. Similarly, correctional institutions and forensic mental health facilities from both language regions in Canada (English and French speaking) were included. Staff members of the Correctional Service of Canada recruited participants from correctional institutions while our research team directly recruited participants from forensic mental health institutions.

As far as recruiting detainees is concerned, we included incarcerated persons who (a) were incarcerated in a Swiss psychiatric or penal institution the time of data collection, (b) were aged 50 years and older, and (c) had at least one contact with mental health services. We excluded participants whose mental state was too instable and/or prison administration did not allow the person to participate for instance due to dangerousness or solitary confinement. Our decision to apply an age cut-off of 50 was due to reasons of “accelerated aging” (for a detailed discussion, see Merkt and Haesen [70]).

### Data collection process

We conducted face-to-face interviews with a purposive sample of (a) incarcerated older persons receiving mental health care in Switzerland and (b) MHPs working with incarcerated patients in Switzerland and Canada. Incarcerated participants were contacted either through a contact person of the prison administration or the mental health service. Expert participants from participating institutions were directly contacted by the research team via email or telephone or by CSC staff.

Study information and informed consent were preventively handed out to the incarcerated participants by our contact persons in those settings and directly sent via email to our expert participants. At the scheduled time and place of the interview, two members of our research group (doctoral students trained in qualitative methodology) explained the purpose of the study, clarified that all data was going to be treated confidentially, and that withdrawal from the study was possible at all times. Thereafter, written informed consent was obtained. There was no compensation provided for study participation. Interviewers and participants met the first time on the day of the interview, thus there was no relationship prior to data collection.

Only one interview-meeting took place with each participant and no repeat interview was conducted. Interviews with incarcerated persons took place within the institutions, but in a specifically provided separate room where conversations could not be overheard. Interviews with experts took place mostly in their office or a location of their choosing. The interviews with the study participants were semi-structured and followed an interview guide specifically developed for the purpose of the project (see Table 1 for an overview of the topics tackled in the interview guides). All interviews were audio-recorded upon the consent of the participant. Field notes were taken during and after each interview. Interviews were held in the language spoken by the participant, either French, English, German or Swiss German. Thereafter the interviews were transcribed verbatim in the language of the conversation. The interviews were checked for the quality and accuracy of the transcriptions, and identifying information was removed. Interview transcripts were not returned to the participants for checking.

**Table 1:** Topic guide for semi-structured interviews

Topic	Patient-participants	Expert-participants
Personal background information	Personal circumstances and social networks (within prison and relationships with outside)	Motivation to work with incarcerated persons, brief description of their work experience and current roles and responsibilities
Aging in the prison context	Relationships with younger persons in detention, satisfaction with work and free time activities offered, perception of prison environment, future plans	Aging in the prison context: exploration of their experiences in working with older patients, prominent therapy topics of older patients
Access to and quality of mental health care	Types of interventions, frequency and duration of treatments, opinion on access to and quality of mental health care, specific aspects of the interventions that helped/impeded therapy progress, perception of their current mental well-being, questions on possible stigma due to mental health issues	Characteristics of care and interaction with older patients, experiences with specific influences due to working in secure contexts (indefinite release dates, dual role conflict—use of elicitation technique, collaboration with other professions and representatives of the justice system)
Risk assessment	Perception of evaluations by forensic experts, experiences with the procedures	Experiences in reporting to the authorities (characteristics, procedures, age as a variable in risk assessments, key criteria in reporting standards)

### Study sample

We conducted a qualitative interview study with 41 older incarcerated persons mandated to treatment and 63 mental health professionals with substantial work experience in secure contexts (see Table 2 for detailed sample characteristics). As we carried out data analysis alongside on-going data collection, we were able to inductively identify when data saturation was reached for each participant group [71], and were able to include more interviewees if we perceived that this was needed. We consider having reached data saturation when the addition of new data does not result in new codes and there is enough information to replicate the study [72].

**Table 2:** Sample characteristics

	Incarcerated older participants	Expert-participants	
		Switzerland	Canada
Time period of data collection	Dec. 2017–Dec. 2018	April 2017–Jan. 2018	Aug. 2017–Nov. 2018
Interview length (in minutes)			
Average	69	71	60
Range	16–120	48–90	28–92
Standard deviation	25.55	14.16	11.49
Number of participants	41	29	34
Participant characteristics			
Gender	2 female	8 female	22 female
	39 male	21 male	12 male
Age	Range 50–76	–	–
	Average 62		
	Standard dev. 6.92		
Language region			
German-speaking	23	16	–
French-speaking	18	13	5
English-speaking	–	–	29
Number of participating institutions	14	14	17
Number of participants per type of institution			
Correctional institution	27	23	21
Forensic-psychiatric institutions	14	6	13

### Context information

In Switzerland, the SCC regulates criminal law at the federal level: sanctions that are imposed are therefore similar across the nation. However, each federal state (canton) is responsible for carrying out the precise execution of the sentences. Thus, some aspects will vary on a cantonal level, such as the settings and placement of mentally ill persons [73].

The SCC distinguishes between penalties (in German “Strafen”) and therapeutic as well as safeguarding measures (“Therapeutische und Sichernde Massnahmen”). Measures are imposed when penalty alone is not sufficient to counter the risk of further offending and the offender requires treatment or treatment is required in the interest of public safety. To impose a measure, courts base their decision on an expert assessment which comprises estimations of (a) the necessity and the prospects of success of any treatment of the offender; (b) the nature and the probability of possible additional offences; and (c) the ways in which the measure may be implemented. Measures are reassessed at regular time intervals and release is granted based on the fulfillment of the requirements of the parole boards and the risk for further felonies.

In our sample, we included persons sentenced to measures according to Art. 59 (in-patient therapeutic measures for mental disorders with the exception of substance use disorders), Art. 63 (out-patient treatment), and Art. 64 (preventative indefinite incarceration) of the SCC. The basic conditions outlined in the previous paragraph concern all measures, but there are further aspects that are specific to each single type of measures. For instance, measures according to Art. 59 and 63 can be ordered if the person suffers from a severe mental disorder that stands in direct connection with the crime committed and it is expected that the measure will reduce the risk to reoffend. Art. 59 requires the person to be confined to a correctional institution of a forensic psychiatric clinic, while a person sentenced under Art. 63 receives ambulatory mandatory treatment. They can either live in the community or be placed in a correctional institution due to an additional penalty. In our sample, we included only persons that were incarcerated at the time of data collection.

Art. 64 can be imposed if an offender is deemed “untreatable”. This form of measure is considered a last resort by legal authorities and courts. Persons under preventative indefinite incarceration do not have to undergo psychotherapeutic nor psychiatric treatment. However, one option to have a prospect of release is to ask for deliberate psychotherapeutic treatment. Treatability is re-evaluated on a regular basis and if it is affirmed, a conversion of the safeguarding in a therapeutic measure is being ordered. Therefore, if a person sentenced under Art. 64 receives mental health care, MHPs have to report to the authorities if the content is of importance to the authorities decision-making process. In our sample, we included persons sentenced under Art. 64 only if they received mental health care.

Concerning the therapeutic settings, in-patient treatment of a measure should ideally be carried out in a psychiatric or therapeutic institution. However, the person can also be incarcerated in a penal institution, given that therapeutic treatment can be provided by specialist staff (e.g. forensic psychotherapists and psychiatrists). The treatment provided will depend on the placement of the person (including the orientation of the institution and the MHP), but also on the type of offense committed and mental health condition. It is therefore not possible to characterise in detail the types of therapies that our participants received. However, it can be said that in practice most persons sentenced to measures will at a minimum receive individual psychotherapy sessions at a regular interval (e.g. weekly, biweekly, or monthly) accompanied by basic (forensic) psychiatric care. Others additionally receive group therapy and some treatment units might apply a “milieu therapy approach”. The type of institution will not give a reliable account of the treatment provided, as, for instance, intense therapeutic treatment units are also available in some penal institutions (see [19] for an overview on placement options for persons sentenced to a measure). This similar context information is also described elsewhere. Please see for example [30, 68].

### Data analysis

The software program MAXQDA was used to support and manage data analysis processes. Initially, four to eight interviews of each participant group were read and coded together by five project members. This allowed the study team to discuss different nuances that were visible in the data and to agree on how to name different codes, and what the codes meant in case of complex code names. Thereafter, four study team members (TW, HS, and collaborators) individually coded all the remaining transcripts using the coded tree that had been developed for each participant group. The coders added new codes when needed. Finally, the team came together to discuss new codes, solve disagreements, and arranged the final coding tree. During the entire process, the team followed thematic analysis [74].

In light of the richness of the data and as a result of the broad scope of the overall interviews, only coded data related to therapist characteristics and activities were extracted and examined for this paper. That is, HS carefully read this sorted data segments in its entirety, examined the codes applied to this data extract, and further analyzed them with the study purpose as the focal point. This topic specific in-depth analysis also followed thematic analysis. Examples of coded quotations were chosen by HS and TW to illustrate the below presented themes. Two research assistants fluent in German, French and English, translated the codes from the original language into English. The translations were then checked by a collaborator and HS, and lastly proofread by an English native speaker. All authors agreed to the results presented in this paper and its interpretation.

## Results

Using data from both older incarcerated persons undergoing court-mandated treatments and experts MHPs, we grouped the results into five themes that all relate to techniques and activities that mental health professionals applied in their work with patients. Not all themes were brought up by both participant groups. Canadian and Swiss expert-participants did not differ systematically in their responses and we therefore do not present them separately. In our results, we indicate Canadian participants as CXX and Swiss experts as SXX, patient-participants are indicated as PXXX. Please see Table 3 for an overview of the themes. In the following sections, we denote the older detainees we interviewed as patient-participants.

**Table 3:** Overview of themes

Themes describing good MHPs	Who described those themes?	Specific characteristics of good MHPs
Treating the patient with respect	Experts and patient-participants	Avoiding stigmatization
		Humanistic approach
Displaying genuine interest in helping the patient	Experts and Patient-participants	Intrinsic motivation to help
		Take the person and his/her issues seriously
		Providing support that exceeds expectations
Recognizing and responding to patients’ needs	Experts and Patient-participants	Incorporating patient’s wishes
		Allowing patient some choices
Tackling topics in detail	Patient participants only	Targeting the important issues
		Identifying underlying emotions
		Taking a different perspective
Perceived skillfulness of the therapists	Patient participants only	Having expertise to respond to therapeutic questions
		Emphasizing positive sides
		Good manners

### Treating the patient with respect: taking a humanistic approach

Expert- and patient-participants emphasized the importance of mental health professionals taking up a respectful attitude towards their patients. This is important in order to avoid labelling the person due to the status as an incarcerated person or based on the crime committed:

“We also have staff in here who think ‘You offender, second class, label, no way’, this is what we have too. I’ve already asked myself, why don’t they quit? If we are that intolerable and it is no fun at all to work with us? Go away. Yes.” (P559)

Some expert-participants described it as a “humanistic idea of man”, according to which one condemns the deeds but not the entire person: “And for us it is really, our idea of man/we are humanistically shaped and we condemn the deeds but not the person.” (S72). In line with this attitude, treating and helping patients to improve their well-being should be at the center of the therapeutic efforts. Respondents that declared following this approach considered developing an understanding of the factors that contributed to the person committing the crime as a central technique during treatment. This in itself can create some relief for the person. At the same time, it has the potential to illustrate the choices made and to exemplify that there are variations in how one can perceive situations and act upon them:

“And that, that it also…the therapist’s, let’s say his interest is to help people to get better. It is of course clear from the beginning that the therapist must clearly state that he does not condemn the person as a human being. That he does not agree with the offence, that he does not have to condemn it because that is what the judge already did. But that he cannot approve this in any way and also ... that it is in any way not about finding reasons for exoneration. Well, on the one hand. But an appropriate understanding of the person who has committed the crime is.” (S68)

Patient-participants seemed to react positively towards this attitude. For instance, the following respondents described how they felt treated as a “regular human being” and at the same time how strongly supported they felt:

“So, she helped me a lot, a lot. And each time when I say to her ‘Thanks a lot, you are my angel’, then she says ‘No, no Ms. (own name), it always comes back to you like the way you are. You may have made a mistake, but you're still a good person.” (P556)

“So you are more as a person than a (inc) to the people here um, but they also knew more about you, your situation, your condition. So um yeah that certainly a/a/at that stage for me it was, was much better, it was far more supportive and you felt the support was there.” (P542)

### Displaying genuine interest in helping the patient

Another basic attitude that patient-participants appreciated was the impression that the MHP had a genuine interest in helping them. Two aspects seemed to contribute to this distinct feeling: first, receiving support that exceeds expectations and that originates from an intrinsic motivation of the MHP to help the patient; and, second, the impression of being taken seriously as person and more specifically perceiving that one’s issues and concerns are taken seriously.

Concerning the first subtheme of a humanistic “support” feeling, interviewees specified that the MHP’s role to provide therapy was more than just an employment. The patient-participants felt that their MHPs were intrinsically motivated to support them: “With her I do not have the feeling, that she is that kind of a therapist who [is like] ‘Yes yes, it is my job, I do therapy, I don’t care about the result, I get my salary anyway.’” (P564). Several expert-participants similarly underlined that it was important that MHP’s motivation should be to help the person: “And that, that it is … the therapist’s, let’s say, interest to help the person to get along better with oneself.“ (S68); and “They are there to work with people and they want to help people” (C37).

This motivation to help consists of a desire to assist the patient in achieving a better future, which goes beyond the mere goal of the justice system for reintegration: “For me, what is important personally, is, in addition to the idea of resocialization, in addition to the protection of victims, also a good future for those affected.” (S27). These basic motives are not only important for the health care personnel, but they should also constitute the overall attitude of the institution:

“I know that both the prison management and the people, who supervise that the sentence is carried out in an adequate way, are people with whom I can collaborate and who are in the same direction as me, even if I don't always agree with it, but I hear, uh, I know that they are also there for the patient's good, for I feel.” (S55)

MHPs who were described as being genuinely motivated to help patients were pictured as persons who provided support that exceeded expectations: “She is a really good woman, she has helped me a lot here, is overly involved, so she really does something, she helps me with problems that come up.” (P554) This notion of support was also prominent in several expert-interviews, as outlined by C41:

“We would often see patients that were admitted here that were in very poor physical condition and when we provided them that physical and mental health care we have seen drastic and marked improvements in their presentation and their quality of life. (…) they um sort of thrive in the supportive environment that we provide and that is very rewarding as well.”

This was also exemplified by other patient-participants, who delineated how MHPs provided help and advice to clarify any issue: “No, really great. Also really awesome, gets involved, makes, looks, does, recommends, takes it apart with you.” (P559). They further characterized this genuine interest by explaining that MHPs would actively listen and provide adequate advice, as succinctly phrased by one respondent: “She listens to me, she gives good advice.” (P544); and: “That is the solution here, listening. And I already feel better, I must say.” (P554).

Respondents described this as feeling to be understood and to receive appropriate advice from the MHP. Patient-participants seemed to make a difference between genuine understanding and simple interest in their well-being. For instance, the following respondent depicted, how s/he perceived one MHP’s statements as inappropriate, because the latter, although concerned about the well-being of the patient, did not fully grasp the patient’s current context and predominant issues:

“Listen, I saw this psychiatrist again. After a while, he started telling me to take care of myself, to take care of myself. That made me so angry, that I stopped seeing him (...) Oh yes, I got angry. ‘Take care of yourself’, [he said]. But I had a child I had to fight for. How do I take care of myself?” (P560)

Other respondents emphasized that listening alone was not sufficient, as highlighted by participant F542: “And I have had this conversation with them and there is no reaction, nothing has changed, reassuring words yes but, yeah I do not know what we can do with words.” In the same vein, this lack of perceived real help affected another interviewee’s motivation to participate in treatment. According to this respondent, therapy lacked a sense of purpose due to the feeling of being left alone to deal with problems: “Sometimes I am not motivated at all, because I sometimes say to myself ‘Why should I talk with them, because yeah, I have to find my own way around, somehow.’” (P535).

Similarly, several respondents highly valued when MHPs’ support went beyond to sole “listening” and reached into practical life support:

“She called the doctor because she noticed that I was always limping. (...) And she helped me to get in shared flat four, that I get a bigger room and to be in open prison. So, she helped me a lot, a lot.” (P556)

Concerning the second subtheme, i.e. the genuine interest in the person, interviewees explained that it was reinforced by a distinct feeling of being taken seriously. This was more specifically described as a sense of one’s concerns and issues being considered, as explained by the following participant: “That’s why I think it’s really good (…) So she takes, she takes the thematic and the problems I have seriously and tries to work on them with me.” (P564) Another respondent specified it as the willingness to take time to listen to the patient and to care for the patient. This happened in the context of receiving support from all professionals involved, of feeling embedded in a team of caretakers:

“Yes, o/ and uh you know that/ that you are taken seriously, we have/we have felt from the beginning that they want time/ yes that they want to take time for you and/and listen and want to help you or, not just ‘Yes, we listen but we will be off work soon anyway, we're leaving". But they, they are there and if there are problems/it is important that a problem also goes to the therapist. Because it's a trio, it's from the occupational/from the occupational therapist, uh reference person, therapist, they work together, don’t they.” (P532)

### Recognizing and responding to patient needs

One aspect that the majority of participants highlighted was the importance to align therapy content with the patients’ interests. Patient-respondents appreciated when they could define the topic of therapy sessions, to address issues that were currently important to them: “It is always only the prisoner who determines the topic of discussion in a therapy session. It is not the therapist who decides ‘Today we do it like this.’” (P544). Expert-participants also highlighted the importance of partly shaping the therapy content along the patients’ interests:

“Content has to be something that they find useful to them - independent of their offending - because a lot of offenders will say to you when you interview them to come into to treatment groups "I do not need this group. I have been convicted of this crime. I have solved all my problems now. I will not do this again in the future" right? It is not how life unfolds (laughs) unfortunately because if every one of them was right I would be out of business.” (C10)

Allowing patients to participate in decision-making on how to design therapy seemed to have an effect on perceived coercion and patient motivation. For instance, the latter example suggests that, by allowing the group to choose the topic of conversation, this person perceived therapy as “less constrained”.

“Then we had a lot of group therapies and they weren’t as coercive ‘you must, you must’, instead Mrs. X sat down, we sat in a circle. And she told us: "Make use of the time." That was her phrase. And then we just started, somebody was talking about something that was bothering him […]. And from these group therapies – [these were] the best conversations.” (P555)

Other interviewees linked this degree in co-determination to their motivation to engage in treatment:

“And then when you are there they say: ‘So now we start.’ (...) and then I say: ‘Yes, but uhm sorry, I've already had two or three days of therapy, therefore.’ and then they say: ‘What counts for us is from now on. That is how our program works, for three to five years, here you go. Do you want to, do you not want to?’ And there the question of motivation is then always a bit, well, okay.” (P538)

The following patient-participant described how the content and the process of treatment was completely against her/his expectations. That is, the MHP did not recognize the person’s current situation and did not adapt the treatment to her/his needs and wishes:

“They started at my birth, so I say "Look, I'm 60, I don't have much time. As soon as there is a place, I have to go to [prison C]. We can't start from childhood on’, [therapist:] ‘Yes, but we cannot rush it.’ ‘Yes, but the psychiatrist has already told me that you can give me advice on how to distract myself in the cell.’ ‘No, we can't do that, [she said]’She had to know why I have claustrophobia … she wanted to talk to my family about why I have this and how they are prepared for the fact that I have to go to prison now. What does that have to do with my claustrophobia? Then I said to her "No, forget it, I'm quitting, I'm not coming back. You can’t help me.” (P556)

In the same line, numerous expert-participants highlighted the importance to allow some choice within therapy. The conditions of court-mandated treatment within prison is predefined, but within this framework there is a certain degree of choice. They emphasized that it was crucial to provide some power in decision-making to motivate patients to actively engage in treatment:

“And, I think especially, I think if we have choices in our whole life, it gives us the will to continue. If you take away the choices of somebody, which in forensics we are taking away their choices to move freely, to move unmonitored. It's controlled.” (C21)

“You know, talk to them about their options. And then of course if they had you know specific referrals, you know we can make them on their behalf. But it is also you know empowering them also. But you know what kind of care they would like to see.” (C35)

Several patient-participants named another reason, why—in their opinion—patients should have a say when determining how to develop therapy sessions: the patient knows best their own deficits and strengths. This was also outlined by several expert-participants:

Above all, I'm going to strengthen the patient in his skills and in everything he knows how to do, and that maybe he forgot that he knows how to do it and how to do it well and probably better than what I could offer him. (S23)

The notion that the patient knows best seemed to be particularly resonating with patients who had previous experience with psychotherapy:

“I told her what I did in institution X and there, I want to continue. And she said that they have a different kind of therapy, how they do it here, how it's organised and all that. And I'm not/I just like had no connection to her either. And she didn't want that/I think as a person I know what I need and what I want. I know it, where I have to make an effort, where I have deficits, what I need.” (P555)

Some expert-participant further emphasized that, while targeting the goals that were important to patients, other issues (such as criminogenic needs) were addressed simultaneously: it used to be that the patient was always last. “And we realized if we start putting their patients' goals first then these other things often also get addressed at the same time.” (C16). Further, a few expert-participants highlighted the need to target patients’ issues in therapy, in the case of patients’ pleading innocence:

“We are no judges. So, hum, we work on the reason why the patient is here, and, and, and, hum, it will hum be his own way to say things. For the patient who is, who is going to claim his, his, his innocence hum for months and months, we will never tell him that he is wrong, in regards to the conviction because it is not our job, but we are going to work on the reason why he is here, because being in prison is not the same thing as being outside, that’s quite clear.” (S23)

### Tackling topics in detail

The majority of patient-participants appreciated MHPs who helped focus the key issues and clarify them in detail. For instance, one patient-respondent that pictured his/her therapist in a positive light stated: “She certainly goes for the/ the living [key issues], that’s for sure” (P535). Participants further emphasized that statements that were too generalized and unspecific were not helpful. The more detailed and specific the MHPs focused on their problematic behavior, the more they perceived treatment as a learning experience. Examples were linked to seeing one’s life and committed crimes from a different perspective, learning how to link emotions to specific thoughts and behaviors, identifying possible offending situations, or learning about the effects their acts can have on victims.

For instance, the following respondent explained how one MHP helped to identify underlying emotions: "She could illustrate it really well to me and then point out the things, that there is a feeling underneath." (P555) In a more general way, this participant described how the MHP helped him/her gain a different perspective on certain aspects of criminal behavior: "And he teased out certain things and worked them out and he pointed stuff out to me that I could not see from my point of view." (P563). Other patient-participants highlighted that they learned from exercises such as recognizing emotions:

"You, you have psychologists or psychiatrists who say: ‘You harm the child with this, with your behavior’. Yes, I don’t have an idea what that means. If you don’t break it down for me: Why do I harm [with] this? What does it trigger? They never did this. And now with this therapist that I currently have, we approach this topic, specifically, (…) Yes, we do/certain/so we have/yes, certain offenses that I committed or so, broken down a little. And then [we] also pursued [this matter] a little and looked at: how did the girl react in this moment? What did she look like from her facial expressions? That’s something I didn’t pay much attention to at that time, because I was in my tunnel, or, uhm yes." (P564)

Moreover, one participant explained how it helped him/her to gain insight into the possible consequences on the victims of the crime committed. To learn about personal life-story of victims helped him/her to understand the impact that a sexual offense can have:

"And then I still had a uhm therapist who worked with victims and he told their cases. And that really unsettled me, of course, yes. Because I did see, … I never resorted to violence in my offenses. But uhm I still, psychologically, and I manipulated, well, but only I never used any physical violence. And there I naturally uhm thought that it was not as bad or something like that. And the victim would have wanted it too …. Because I got the insight into the victim’s situation and the thing with empathy and all, got me a totally different picture and of course I saw all my offenses from a totally different perspective." (P565)

The same respondent explained how s/he learned how to identify situations in which s/he might reoffend and how this helps to plan free-time activities accordingly: "I know what I am allowed to do, where I go and during holidays: what do I do on vacation? Uhm especially where there are children, if I go to an event where there are children and so forth, that I don’t do that anymore" (P565).

### Perceived skillfulness of therapists

Last, the skillfulness of MHPs was raised by several patient-participants, the majority of such references being linked to negative treatment experiences. The behaviors that were linked to perceiving MHPs as unqualified were the tendency by MHPs to look only for the negative sides of a patient, their inability to respond to therapeutic questions, their lack of good manners, or a perceived helplessness of the MHP when not knowing how to help the patient. For instance, one participant named reasons why s/he perceived the MHP as insufficiently qualified: "When I asked something therapeutic or something, then she googled it, then she had to look it up (…) yes just unexperienced, she did not come across like a therapist." (P532) However, a certain degree of reflectiveness and questioning oneself was highly valued:

"And she can question herself that is maybe also of some importance. I only experienced this very rarely. Do this and your patients will appreciate you! In case you are going into forensics [prison mental health care]. (I laughs) But I also don’t think that this is wrong otherwise, when one can question oneself and can say “Oh, I underestimated that”, something of importance or anything." (P533)

Other patient-participants pictured insufficiently qualified MHPs as people who, in their opinion, did not show good manners. For instance, they described the persons as loud and not very eloquent: "When I see employees who express themselves like peasants and Neanderthals and behave like it and stamp and yell through the whole unit, then I wonder how they got this job." (P559). Another interviewee summarizes his/her desperation in a way that s/he had the impression, that some MHPs were ‘at the end of their rope’, that they seemed overcharged with his/her case and did not know what to do:

“P: Yeah, it was it was giving the drugs, it was escalation, I kept saying to, you know, to them that "I am getting worse. I am getting worse. I am getting worse". Ahm and I just do not think they necessarily knew what to do. Hum I think that was the fundamental problem.” (P542)

## Discussion

Our study findings provide valuable insights into therapist characteristics and activities that facilitate change in correctional contexts. This is, to our knowledge, the first study to combine qualitative data from Swiss and Canadian experts and to integrate those with the experiences of detained patients mandated to treatment. Our study provides an important contribution to psychotherapy process research, specifically targeting court-mandated treatment settings, for which little data is currently available. Thus, our results contribute to much-needed empirical evidence to improve patient’s adherence in court-mandated treatment orders.

Above all, our results underline the importance of positive and respectful attitude in the provision of care in the forensic setting. This attitude is best represented by showing genuine interest in supporting the patient, by taking the time to talk and by demonstrating active listening skills, followed by adequate and tangible advice or help. Second, our findings highlight the significance of the MHPs’ willingness and ability to understand the patients’ particular needs and their overall context. By allowing patients to raise their personal concerns and therefore granting them some choice and control over the content and course of therapy, a positive message is delivered to forensic patients, i.e. that they are valued and respected. Further, patient-participants highlighted that it is central to focus on their issues in detail and link treatment to very specific and concrete life examples to progress in therapy and to advance their understanding of the crime committed. Lastly, the MHP’s qualification and skillfulness was questioned when they were not able to answer technical questions and gave the impression of being at a loss with therapeutic options.

Our results support previous claims that it is crucial to face incarcerated patients with a respectful and genuine attitude (see for example [46, 48]). The participants may have underscored these attitudes due to their status as prisoners and fear of being discriminated due to this label. They are frequently subject to labelling attitudes and with self-esteem likely to be low among this group, it makes them susceptible to humiliation [75, 76]. Taking up a respectful attitude is also important due to the relation of low self-esteem with violence [75] and its potential to impede change [77]. Moreover, respect is not exclusive to this context, as studies in general psychiatry also underline the influence of understanding and respecting patients with dropout rates and outcome measures [78]. Respectful attitudes can be described as a therapist treating the individual as a person of worth, which is linked to the overarching concept of “positive regard” [10]. However, what is specific to offender treatment is to show an accepting attitude by separating the patient as a person from his/her offense [79]. This approach was confirmed by our participants, who underlined the value of a *“humane approach”.*

Along the same lines, displaying genuine interest in treating and helping patients might contribute to a positive and respectful attitude. Participants delineated positive therapy experiences when therapists took the extra time to talk and to listen actively, provided adequate advice or concrete support, and took patients and their problems seriously. By doing so, patients perceived therapists as genuinely concerned about their patients, which could likewise contribute to positive regard. Positive regard is one of the fundamental therapist characteristics, as delineated by Rogers [80]. While there is no universally accepted definition of this term, positive regard is frequently described with concepts such as support, affirmation, respect, validation, and active listening. As these and similar terms were frequently expressed by our respondents to describe good MHPs, it might therefore implicate that the overall concept of positive regard is vital in the work with incarcerated persons.

The prominence of positive regard, however, also raises the question whether it is particularly difficult for MHPs to display warmth and maintain a positive attitude towards patients who have committed a crime. For instance, Harris, Happell [81] showed that patients’ crimes elicited fear and disgust in forensic nurses. To cope with their anxiety, the nurses distanced themselves emotionally and held negative attitudes towards their patients. In general, working with people who offended is described as mentally, physically, and emotionally draining [82]. It is difficult to maintain hope, in particular with patients who are detained long-term [83]. Nevertheless, some MHPs described it as a challenging but rewarding work, and this “*humane attitude*” mentioned above can also serve as a coping strategy for MHPs to manage their own negative responses towards patients’ offensive acts [82]. Further, as Farber, Suzuki [10] emphasize that one’s ability to show positive regard towards a patient should be regular topic of supervision, it seems to be crucial when working with people who offended.

Moreover, it is important to allow a certain level of choice and control over the topic and content of therapy to also target criminogenic factors. This, as some experts emphasized, can be implemented by fulfilling patients’ needs and wishes. Such expert opinions seem to mirror current developments in the treatment of incarcerated populations. In the past two decades, the focus from targeting exclusively criminogenic factors shifted towards the incorporation of more resource-oriented approaches into offender treatment [84] such as the Good Live Model (see for example [85]). The patient’s perspective showed that they did appreciate some control concerning the therapy content, which they linked to their motivation to participate, as well as to less perceived coercion. The issue of experiencing some degree of control within a very restrictive environment has been broached by other qualitative research with older forensic patients [64]. Even just for reasons of engaging the patient into treatment, it seems valuable to allow the patient to co-determinate therapy content (see for example [36, 37]). Similarly, Hachtel and Vogel [33] concluded that patients’ want to be respected and participate in the decision-making process.

Another therapist technique that was vastly appreciated was the ability to target personal issues. That is, it was discussed that it is not enough to take time to focus on the critical issues, but it is also necessary to support patients in linking thoughts and feelings with specific behaviors. Respondents valued when therapists helped them break down questions and issues, and provided specific advice and concrete examples, while avoiding vague comments and abstract explanations. This can be closely linked to the directiveness concept proposed, for instance, by Marshall and Serran [13]. The latter revealed that it was beneficial when therapists suggested possible directions or alternatives to patients struggling with issues. It is further striking that our expert-participants did not discuss this topic. Reasons might be either that they are not aware of this issue or that it is self-evident. Nevertheless, this emphasizes the importance that MHPs adopt the use of detailed and concrete explanations. Our results therefore provide an important contribution on how MHPs should target sensitive issues, such as past crimes committed.

Lastly, it is questionable in what relation the perceived skillfulness of MHPs stands in connection to their actual qualification. The example mentioned in the results—in which a participant explained that one of his/her former treating therapists was not able to respond to therapeutic questions without using “Google” as a help—could be linked to clinical experience and expertise. Indeed, some studies show a slight positive correlation between psychotherapist’s expertise and clinical experience with outcome factors [86]. As our respondents also linked poor qualification with aspects such as bad manners, issues other than qualification might lead to this overall judgement. For example, general frustration and dissatisfaction with the mental health care received might result in a person describing the MHP as poorly qualified. MHPs should therefore observe a patient’s treatment satisfaction carefully to counteract negative feelings towards therapy. Such vigilance might be particularly important, as our expert-participants did not elaborate on this topic, suggesting that it is not at the center of their attention. Issues such as a patient’s treatment satisfaction as well as MHPs general self-presentation on the treatment unit should therefore be monitored closely.

### Limitations

Incarcerated respondents might have participated due to reasons related to coercion: indeed, participation in our research study might have been encouraged by other players or by the hope (in spite of the information provided by the research assistant during recruitment) that it could increase their chances for release. This potentially impacts reliability and validity of study results as participants might have brought an agenda to the interview setting [87, 88]. We tried to limit the influences of coercion and social desirability by conducting the interviews in a private atmosphere (separate room in the correctional institution in which conversations could not be overheard) and by assuring anonymity during the whole process of data analysis. We emphasized the voluntary nature of participation before starting the interviews and explained the purpose and conditions of our research project thoroughly before the interview. Participants were informed that they could refuse participation and that information will not be communicated to avoid any negative consequences. Since our results contain narratives of positive and negative treatment experiences, we have the impression that participants felt free and safe to talk without constraints.

Moreover, stakeholders that were interested in participating might have had a specific set of opinions that influenced the study results. As with the older incarcerated participants, we assured anonymity and confidentiality to limit the influence of social desirability. Nevertheless, institutional regulations and cultural mindsets that prevail in a certain environment might have affected their attitudes towards discussing what is important when working with court-mandated patients. Our participants worked in diverse settings (forensic units and prisons) and in different countries across several language regions (two language regions in Switzerland and two in Canada) and therefore might have advanced opinions that exist in the respective contexts. However, our study provides insight into overarching activities that are important in a wide range of court-mandated settings.

## Conclusions

Psychotherapeutic encounters in court-mandated treatment settings are challenged by coercion and control. Our findings show that MHPs working with patients legally referred to treatment need to put additional focus on displaying a respectful and positive attitude. A particular strategy to do so was described as the *“humane approach”*, i.e. the capacity to value the persons under treatment and to separate them from the offensive acts previously committed. Such attitude can make patients feel respected and might at the same time contribute to more positive feelings on the MHPs’ side. Furthermore, in light of the coerciveness of the therapy context, our study highlighted that it is of particular importance to grant patients some degree of choice and control over the content and course of therapy. Doing so will engage and motivate the patient to actively participate in treatment. In addition, feedback and advice needs to be concrete, detailed, and applied to the person’s current situation. Vague and general comments need to be avoided. Lastly, patients questioned the MHPs’ qualification when they did not progress in therapy. MHPs should therefore monitor patients’ treatment satisfaction carefully to counteract negative feelings towards treatment participation. In sum, while some therapist activities that promote change in psychotherapy might be similar to general psychiatry patients—such as positive regard—the way they are established might differ slightly in a coercive context. Our study therefore contributes to much-needed research on therapist characteristics that are specific to these contexts. Based on our study findings, we suggest the following for forensic practice and research.

1. Build a treatment setting where negative feelings towards patients are monitored regularly—through, for example, supervision and peer consulting—in order to develop strategies such as the “humane approach” to handle negative emotions.
2. Monitor patient motivation and engagement in treatment by ascertaining their satisfaction with treatment, understanding their wishes and needs, and clearly communicating with them.
3. Future research should examine the effectiveness of identified therapist characteristics and activities in improving treatment provided to forensic patients. For instance, such studies could assess whether expressions that are linked to the “humane approach” (condemning the offensive acts but valuing the human being) are correlated to a therapist’s ability to form therapeutic alliance and if it is directly related to reducing recidivism.
4. Last, future research could use comparative quantitative approach to study the relationship between therapist characteristics and activities in criminal court-mandated settings and treatment motivation as well as treatment adherence amongst both younger and older incarcerated adults. This, to highlight possible discrepancies between the age groups but also to assess concepts that are equally important to all age groups.

## Data Availability

The dataset analyzed during the current study is not publicly available. Our analysis is based on qualitative interviews with mental health professionals working in secure settings and older persons living in detention. The individual privacy of our study participants would be compromised if we shared the whole transcripts publicly. However, we can share the parts of the transcripts relevant for this paper upon reasonable request. Please contact either tenzin.wangmo@unibas.ch or helene.seaward@unibas.ch.

## Abbreviations

MHP: Mental health professional
SCC: Swiss criminal code
